# Antibody conjugation of paclitaxel enables aqueous compatibility and enhances tumor-targeted efficacy *in vitro* and *in vivo*

**DOI:** 10.3389/fphar.2026.1805542

**Published:** 2026-03-23

**Authors:** Gul E. Rana, Shufan Yang, Yanting He, Chengcheng Qiu, Jing Long, Yezhen Huang, Wenjing Liu, Jincheng Luo, Jindi Zhu, Siwen Xu, Qi Wang, Chun-He Wang

**Affiliations:** 1 Biotherapeutics Discovery Research Center, Shanghai Institute of Materia Medica, Chinese Academy of Sciences, Shanghai, China; 2 University of Chinese Academy of Sciences, Beijing, China; 3 Joint National Laboratory for Antibody Drug Engineering, School of Medicine, Henan University, Kaifeng, China; 4 Wuya College of Innovation, Shenyang Pharmaceutical University, Shenyang, China; 5 School of Life Science and Technology, China Pharmaceutical University, Nanjing, China; 6 New College of Traditional Chinese Medicine, Nanjing University of Chinese Medicine, Nanjing, China

**Keywords:** antibody-drug conjugate, *in vivo* efficacy, paclitaxel, solubility, tumor targeting

## Abstract

**Introduction:**

Paclitaxel (PTX) remains a widely used chemotherapeutic agent for treating diverse solid tumors, but its clinical utility is constrained by pronounced hydrophobicity, which necessitates the use of formulation surfactants and contributes to a narrow therapeutic window. To overcome these limitations, we developed a TROP2-targeted antibody-drug conjugate (ADC) designed for selective and solvent-free delivery of PTX.

**Methods:**

The ADC, designated hRS7-PTX, was generated by conjugating PTX to the humanized anti-TROP2 monoclonal antibody (hRS7) through a rationally engineered, PEGylated, protease-cleavable linker. The conjugate was characterized for its physicochemical stability, solubility, and antigen-dependent cytotoxic activity *in vitro*. *In vivo* efficacy and biodistribution were evaluated in murine xenograft models of TROP2-expressing tumors.

**Results:**

hRS7-PTX demonstrated high aqueous stability and maintained favorable physicochemical properties without the need for organic co-solvents. *In vitro*, the ADC induced potent, antigen-dependent cytotoxicity, preferentially killing TROP2-positive tumor cells while sparing antigen-negative cells. In murine xenograft models, hRS7-PTX treatment produced sustained tumor regression and significantly outperformed weight-equivalent doses of conventional PTX formulations. Fluorescence imaging further confirmed rapid and preferential accumulation of hRS7-PTX within TROP2-rich tumor tissues.

**Discussion:**

These findings establish hRS7-PTX as an effective, solvent-free strategy for targeted PTX delivery that expands the therapeutic index of this widely used drug. The combination of selective tumor targeting, improved tolerability, and robust antitumor activity supports the further development of hRS7-PTX for the treatment of TROP2-positive malignancies.

## 1 Introduction

Paclitaxel (PTX) is a widely used chemotherapeutic agent and a key component of treatment regimens for breast, ovarian, lung, and other solid tumors (Nawara et al., 2021). Its anti-tumor activity is mediated through stabilization of microtubules, leading to disruption of mitosis and induction of apoptotic cell death (Wang et al., 2000). Despite its broad clinical efficacy, this natural compound lacks ionizable functional groups, resulting in poor aqueous solubility (<1 μg/mL) (Yang et al., 2015). Owing to its poor aqueous solubility, conventional paclitaxel (PTX) formulations rely on organic solvents and surfactants, most notably Cremophor EL® with dehydrated ethanol. The Cremophor EL®-based formulation (Taxol®), approved in 1992, has been widely used as a frontline chemotherapeutic agent; however, Cremophor EL® has been linked to hypersensitivity reactions, altered drug disposition, and dose-limiting toxicities, which limit optimal therapeutic dosing (Liu et al., 2015; Lal et al., 2009). In addition, the non-specific biodistribution of free paclitaxel contributes to systemic toxicity and limits the achievable therapeutic index (Austria et al., 2025).

To overcome these challenges, several formulation-based strategies have been developed to improve the apparent solubility and tolerability of paclitaxel. These include albumin-bound nanoparticles, liposomal formulations, polymeric micelles, and prodrug approaches (Lee et al., 2020; Yang et al., 2007; Chen et al., 2020). While such systems reduce solvent-related adverse effects and enhance formulation stability, they largely rely on passive tumor accumulation mechanisms and do not fully overcome the lack of tumor-selective drug delivery. As a result, off-target exposure remains substantial, particularly at higher doses, highlighting the need for alternative strategies that enable both improved formulation properties and targeted delivery (Chehelgerdi et al., 2023). However, while nanoparticles offer substantial improvements in drug delivery, issues such as tumor heterogeneity, poor tumor penetration, and non-specific toxicity persist, thereby limiting their widespread clinical use (Shin et al., 2026; Munir, 2022; Su and Hu, 2018).

Antibody-drug conjugates (ADCs) have emerged as a unique therapeutic modality that integrates the high specificity of monoclonal antibodies with the cytotoxic activity of small-molecule drugs. By selectively binding tumor-associated antigens and undergoing receptor-mediated internalization, ADCs preferentially deliver cytotoxic payloads to antigen-positive malignant cells, thereby reducing non-specific exposure of healthy tissues that do not express the target antigen (Chen et al., 2025; Wang et al., 2025; Fong et al., 2025). To date, the majority of clinically approved ADCs employ highly potent payloads such as auristatins and maytansinoids, which are effective at sub-nanomolar concentrations but are often associated with narrow therapeutic windows and toxicity concerns (Drago et al., 2021; Nguyen et al., 2023). In recent years, there has been a rapid expansion of ADCs using moderately potent topoisomerase I inhibitor payloads, particularly camptothecin derivatives such as deruxtecan and SN-38, exemplified by trastuzumab deruxtecan and sacituzumab govitecan. However, beyond this topoisomerase I inhibitor class, the use of other clinically established, moderately potent chemotherapeutic agents, such as taxanes as ADC payloads remains relatively limited, with only a small number of paclitaxel-based ADCs reported and none yet reaching clinical approval, especially with respect to systematic studies on how conjugation impacts their aqueous compatibility and formulation behavior (Valsasina et al., 2024; Pommier and Thomas, 2023).

Paclitaxel represents an attractive yet challenging candidate for ADC development. Its clinical relevance, well-understood mechanism of action, and proven antitumor efficacy make it a compelling payload; however, its hydrophobicity and bulky molecular structure complicate conjugation, formulation, and stability. Advances in linker chemistry have renewed interest in expanding ADC payload diversity (Wang et al., 2022; Wang et al., 2017). Cleavable dipeptide linkers such as valine-citrulline (VC) enable efficient intracellular drug release following lysosomal protease cleavage and have been adapted, together with PEGylated motifs, to generate paclitaxel-based ADCs with improved *in vivo* efficacy and tolerability, including TROP2-targeting constructs such as hRS7-VK-PTX (McCombs and Owen, 2015; Yajaman et al., 2025; Shao et al., 2020). Despite these advances, prior work has primarily focused on antitumor activity and safety. In contrast, the extent to which antibody conjugation fundamentally alters the aqueous compatibility and practical formulation behavior of paclitaxel itself has not been systematically investigated.

Beyond targeted delivery, antibody conjugation has the potential to fundamentally alter the physicochemical behavior of hydrophobic small molecules. By covalently attaching paclitaxel to a large, water-soluble antibody scaffold, it may be possible to functionally overcome paclitaxel’s intrinsic aqueous incompatibility, enabling formulation in physiologically relevant buffers without the need for organic co-solvents or surfactants. However, systematic investigations that relate antibody conjugation to both aqueous compatibility (e.g., turbidity/aggregation behavior in simple buffers without co-solvents) and biological performance of paclitaxel remain limited (Ebrahimi et al., 2023; Buecheler et al., 2020; Singh et al., 2015).

**FIGURE 1:**
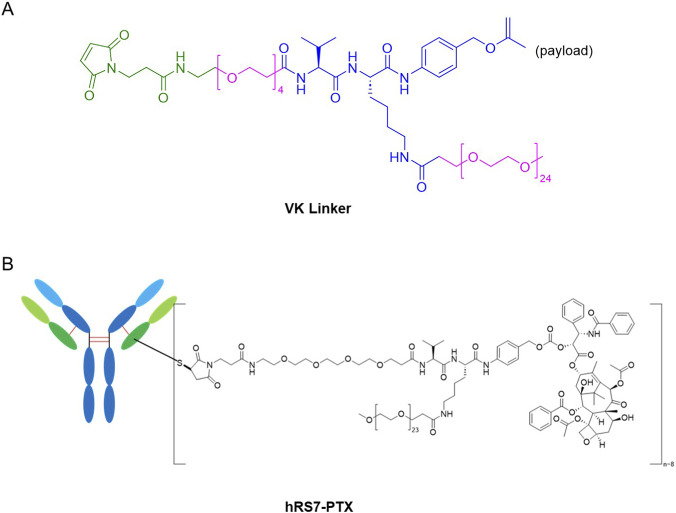
**(A)** Chemical structure of the PEGylated VK linker-payload used in this study, highlighting the PEG segments (pink) and thiol-reactive maleimide moiety (green). **(B)** Schematic representation of hRS7-PTX showing conjugation of the VK linker-payload to reduced cysteines on hRS7.

In this study, we systematically evaluate and characterize a TROP2-targeting paclitaxel-based antibody-drug conjugate constructed using a PEGylated cleavable dipeptide linker (Figure 1). We comprehensively analyze its physicochemical properties, drug-to-antibody ratio, purity, and stability, with particular emphasis on aqueous behavior in PBS without formulation additives. Furthermore, we compare the *in vitro* cytotoxicity, cellular internalization, and *in vivo* antitumor efficacy of the paclitaxel ADC with free and a minimally formulated paclitaxel control.

Our results demonstrated that antibody conjugation functionally overcomes the aqueous incompatibility of paclitaxel and significantly enhances its tumor-targeted therapeutic performance *in vivo*. These findings support the feasibility of paclitaxel-based ADCs as a promising strategy for improving the delivery and efficacy of this clinically important chemotherapeutic agent.

## 2 Materials and methods

### 2.1 Cell lines and reagents

BxPC-3, HEK293, and NCI-H1688 cell lines were sourced from the Shanghai Cell Bank (Shanghai, China). Cells were cultured in RPMI 1640 or DMEM medium supplemented with 10% fetal bovine serum (FBS) and maintained at 37 °C in a humidified incubator with 5% CO_2_. The Cell Counting Kit-8 (CCK-8) was obtained from Dojindo Laboratories (Tokyo, Japan). Matrigel matrix was purchased from Biocoat. Paclitaxel (PTX) powder was purchased from Meilun Bio and Tris (2-carboxyethyl)phosphine (TCEP) was obtained from Pierce.

### 2.2 Antibody reduction and conjugation

The antibody concentration was adjusted to 10 mg/mL and reduced with 8 M equivalents of TCEP for 2 h at 37 °C. After cooling at room temperature for 10 min, 12 M equivalents of the drug-linker were added, and the reaction proceeded at 25 °C for 30 min. Unreacted drug-linker was removed by ultrafiltration, and the ADC was resuspended in PBS. ADC concentration was determined using a Nanodrop spectrophotometer.

### 2.3 SDS-PAGE

Samples (3 µg) were mixed with 5× loading buffer, heated at 100 °C for 10 min, and separated using a 10% SDS-PAGE gel. Gels were stained using Coomassie Brilliant Blue and images were captured with a ChemiDoc MP Imaging System (Bio-Rad).

### 2.4 SEC-HPLC

SEC-HPLC was done using a Thermo Scientific HPLC (High-Performance Liquid Chromatography) system with a MAbPac SEC-1 column (5 μm, 7.8 × 300 mm; P/N 088460, Thermo Fisher Scientific). PBS served as the mobile phase with the column maintained at 25 °C and a flow rate of 1 mL/min. Detection was carried out at 280 nm with an injection volume of 15 μL.

### 2.5 RP-HPLC analysis

RP-HPLC was done using an Agilent HPLC system equipped with a PLRP-S 1000Å, 8 μm, 4.6 × 250 mm column (Agilent Technologies). Mobile phase A consisted of water with 0.1% TFA, and mobile phase B consisted of acetonitrile with 0.1% TFA. The gradient program was set as follows: 0–3 min, 100% A; 3–25 min, linear gradient from 100% A to 100% B; 25–30 min, 100% B. The column temperature was set at 70 °C, and a 10 μL sample was loaded for injection.

### 2.6 LC-MS analysis

LC-MS analysis was done using a Waters Xevo G2-XS QTof mass spectrometer coupled to an ACQUITY UPLC system using a Waters Protein BEH C4 column (1.7 μm, 2.1 × 50 mm). Mobile phase A consisted of ultrapure water containing 0.1% formic acid, and mobile phase B consisted of acetonitrile containing 0.1% formic acid. The flow rate was set to 0.3 mL/min, and the column temperature was maintained at 80 °C. The gradient program was as follows: 0–2 min, 5% B; 2–6 min, linear gradient from 5% to 90% B; 6–7 min, 90% B; 7–7.5 min, 5% B; 7.5–8 min, 5%–90% B; 8–8.5 min, 90%–5% B; and 8.5–10 min, 5% B. Samples (1 mg/mL) were deglycosylated with PNGase F at 37 °C for 30 mins, reduced with TCEP at 37 °C for 15 min, filtered through a 0.22 μm membrane, and injected at 10 μL. Mass spectra were acquired in positive ion mode over an m/z range of 500–2500, and DAR values were determined by spectral deconvolution using UNIFI software.

### 2.7 Aqueous compatibility and solubility assessment

PTX and ADC formulations were directly dissolved in PBS (pH 7.4) without adding organic solvents or surfactants and incubated at 4 °C and room temperature. Samples were assessed at 1, 6, 12, 24, 48, 72, 120, and 168 h. At each time point, solutions were visually inspected for precipitation or turbidity, and optical density (OD) at 600 nm was recorded to quantify solution clarity. Experiments were performed in triplicate, and both visual observations and OD 600 readings were used to evaluate aqueous compatibility. The turbidity was measured using the following formula: Turbidity (OD_600_) = OD_600, sample_−OD_600, blank_.

### 2.8 Flow cytometric analysis of TROP-2 expression and binding

Cells were incubated with antibody, ADC, an isotype control, isotype-ADC, and blank at various concentrations ranging from 300 nM and serially diluted two-fold in ice-cold staining medium on ice for 30 min. After incubation, cells were washed twice with ice-cold staining medium to eliminate unbound antibodies. Cells were then stained with PE-conjugated goat anti-human IgG (5 μg/mL) in ice-cold staining medium for 30 min on ice and washed twice. Fluorescence intensity was assessed using flow cytometry, and the data were used to generate binding curves for TROP-2 and to assess the effects of the different antibody and conjugate treatments.

### 2.9 Cell viability assays

Cells were seeded in 96-well plates at a density of 5,000-10,000 cells per well and allowed to adhere overnight. Gradient-diluted drugs were added, and cells were incubated for 96 h at 37 °C in a humidified incubator with 5% CO_2_. Next, 10% CCK-8 reagent was added to the medium and incubated for 1–2 h, after which the absorbance at 450 nm was recorded using a SpectraMax M5e microplate reader (Molecular Devices). Cell growth inhibition was calculated relative to untreated control cells. Dose-response curves were plotted based on the mean of triplicate measurements, and IC50 values were determined using Prism GraphPad Software.

### 2.10 Internalization assay

Cells were incubated with antibodies and ADCs on ice for 30 min. After washing, fresh complete medium was introduced, and the cells were incubated at 37 °C with 5% CO_2_. At designated time points, cells were incubated with anti-human IgG-PE secondary antibody for 30 min. Changes in PE fluorescence intensity were measured using flow cytometry to determine the internalization rate.

### 2.11 Mouse xenograft models

Five-week-old NOD/SCID mice were obtained from Charles River Company (Shanghai, China). All animal procedures complied with IACUC guidelines of the Shanghai Institute of Materia Medica, CAS. BxPC-3 cells were re-dispersed at a concentration of 5 × 10^6^ cells/mL in PBS containing Matrigel and were injected subcutaneously into the left axillary region of each mouse. Once tumors reached approximately 170 mm^3^, mice were randomly allocated to treatment groups. Drugs were administered via intravenous injection at varying doses twice a week. Tumor volumes and mouse body weights were measured, with tumor volume calculated as: V = (length × width^2^)/2. Toxicity associated with different treatment group was evaluated by monitoring the body weight loss.

### 2.12 *In vivo* fluorescence labeling and biodistribution analysis

ADC and Iso-ADC were adjusted to a concentration of 1 mg/mL, and the buffer was exchanged to PBS (pH 7.4). The near-infrared fluorescent dye DyLight™ 800 NHS Ester was solubilized in deionized water to achieve a final concentration of 1 mg/mL, aliquoted, and kept at −20 °C protected from light until use. The appropriate amount of fluorescent dye was calculated based on the molar ratio between antibody and dye and added to the antibody-drug conjugate solutions. The reaction mixtures were gently mixed and incubated at room temperature for 1 h, protected from light. The amount of dye added was calculated according to the following formula:

Mass of dye = (Mass of protein/Molecular weight of protein) × 10 × Molecular weight of dye. Following the labeling reaction, samples were transferred to 10 kDa molecular weight cut-off ultrafiltration devices and centrifuged at 4,000 rpm for 5–10 min at 4 °C to remove unreacted dye. The retained conjugates were collected, adjusted to a final concentration of 1 mg/mL, and stored at 4 °C protected from light until further use. Tumor-bearing mouse models were established by subcutaneous inoculation of BxPC-3. When tumors reached an appropriate size, mice were randomly allocated to the experimental groups and intravenously injected via the tail vein with 200 µL of fluorescently labeled ADC or Iso-ADC. At 0, 24, 48, and 72 h post-injection, mice were anesthetized using inhaled isoflurane, and whole-body fluorescence imaging was performed using a small-animal *in vivo* imaging system to monitor the biodistribution and tumor accumulation of the conjugates. Fluorescence images were acquired under identical conditions for all groups, and signal intensities at tumor sites were analyzed to evaluate tumor-targeting efficiency.

### 2.13 Dose tolerability assay

Dose tolerability assay was conducted in five-week-old NOD/SCID mice (3 mice per dose). Drug was administered as a single intravenous injection at 100, 150, and 200 mg/kg for ADC. Toxicity was assessed by observing mouse behavior, weight loss, and survival. The percentages of change in body weight (%) was calculated as: (body weight-body weight before treatment)/(body weight before treatment) ×100%.

### 2.14 Statistical analysis

Data were processed using GraphPad Prism 8.0 (GraphPad Software Inc.). P-values were calculated via two-way ANOVA with multiple comparisons (*P < 0.05, **P < 0.01, ***P < 0.001, ****P < 0.0001; ns: not significant).

## 3 Results

### 3.1 Structural integrity, homogeneity, and stability of hRS7-PTX ADC

The hRS7 monoclonal antibody was successfully conjugated to paclitaxel (PTX) using a cysteine-targeted conjugation strategy to generate the hRS7-PTX antibody-drug conjugate (ADC). In brief, the antibody was partially reduced with tris(2-carboxyethyl)phosphine (TCEP) to selectively expose free thiols on solvent-accessible cysteine residues. The reduced antibody was subsequently reacted with the PTX-VK linker at defined molar ratios under controlled conditions, resulting in covalent attachment of PTX molecules to the antibody scaffold. Following conjugation, unreacted drug-linker was efficiently removed via ultrafiltration, and the ADC was redispersed in PBS for comprehensive physicochemical characterization.

**FIGURE 2:**
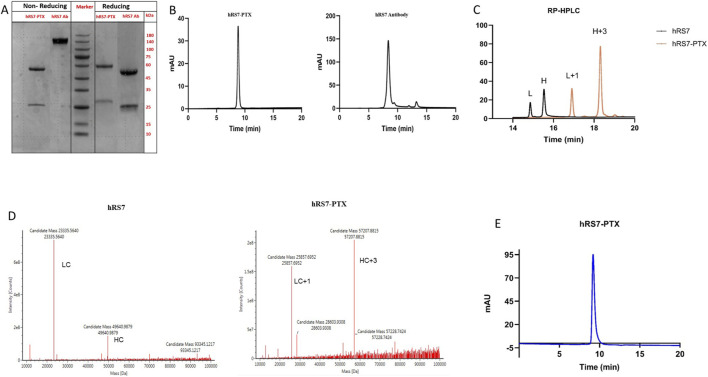
**(A)** SDS-PAGE under reducing and non-reducing conditions shows expected molecular weight shifts after PTX conjugation, with no fragmentation, and preserved inter-chain disulfide linkages. **(B)** Size-exclusion HPLC (SEC-HPLC) analysis of hRS7-PTX ADC (left) and unconjugated antibody (right). Chromatograms show a dominant monomeric peak for both samples, indicating that conjugation did not compromise antibody integrity or induce nonspecific aggregation. **(C)** Reverse-phase HPLC (RP-HPLC) analysis comparing hRS7-PTX ADC (orange) with unconjugated antibody (black). RP-HPLC shows increased hydrophobic retention of hRS7-PTX, confirming PTX incorporation and removal of free drug. **(D)** LC-MS analysis of deglycosylated and reduced hRS7-PTX ADC (right) versus unconjugated antibody (left) confirms mass increases corresponding to PTX conjugation and a DAR of 8. **(E)** SEC-HPLC after 30 days at 4 °C demonstrates stable monomeric hRS7-PTX with no aggregation or degradation.

SDS-PAGE analysis conducted under denaturing conditions revealed clear upward shifts in both the heavy chain (HC) and light chain (LC) of the ADC compared with the unconjugated antibody, with the LC around 25 kDa and the HC around 50 kDa, consistent with successful attachment of PTX-linkers (Figure 2A). No additional lower-molecular-weight species or degradation products were observed, indicating that the conjugation process preserved antibody integrity. Under non-reducing conditions, the intact hRS7 antibody exhibited an approximate molecular weight of 150 kDa. For the ADCs, conjugation of thiols in the hinge region with the linker-drug resulted in two prominent bands corresponding to the light and heavy chains. Overall, the SDS-PAGE results confirmed that the molecular weights of the ADCs were as expected and that no significant additional bands were present, demonstrating the high purity of the conjugated samples.

Size-exclusion HPLC (SEC-HPLC) analysis showed a dominant monomeric peak for the ADC, with no evidence of high-molecular-weight aggregates or low-molecular-weight fragments (Figure 2B). These findings indicate that conjugation did not induce nonspecific aggregation or destabilization of the antibody scaffold, and that the ADC retained its monomeric state, which is critical for predictable *in vivo* behavior.

Reverse-phase HPLC (RP-HPLC) analysis revealed increased hydrophobic retention of the ADC relative to the unconjugated antibody, reflecting the incorporation of the hydrophobic PTX payload (Figure 2C). Shifts in retention times were consistent with site-specific attachment, and ultrafiltration effectively removed any residual free PTX, confirming purity and proper conjugation.

LC-MS analysis of deglycosylated and reduced ADC samples was conducted to verify chain-specific drug conjugation and molecular composition. The theoretical molecular weight of the VK-PTX linker is 2522.89 Da and was used for interpretation of the observed mass shifts. The unconjugated light chain (LC) exhibited a molecular mass of 23,335.5640 Da. Following conjugation, the predominant LC species was detected at 25,857.6952 Da, corresponding to a mass increase of 2522.13 Da, which is consistent with the theoretical mass of a single VK-PTX linker, indicating mono-substitution of the light chain. The unconjugated heavy chain (HC) showed a molecular mass of 49,640.9879 Da. In the conjugated ADC sample, a major HC peak was observed at 57,207.8815 Da, representing a mass increase of 7566.89 Da. This increase closely matches the expected mass addition for three VK-PTX linker molecules, confirming tri-substitution of the heavy chain. Taken together, the LC-MS results demonstrate a defined and reproducible conjugation profile, with one VK-PTX linker on each light chain and three on each heavy chain. Consequently, the intact antibody contains eight PTX molecules, corresponding to a mean drug-to-antibody ratio (DAR) of 8 (Figure 2D), consistent with the designed ADC architecture.

Stability studies indicated that the ADC retained its structural integrity and monomeric profile when stored at 4 °C in PBS for 30 days. SEC-HPLC analysis after this period showed no detectable aggregation or degradation, confirming that the ADC is chemically and physically stable under physiologically relevant storage conditions (Figure 2E).

Collectively, these data demonstrate that the hRS7-PTX ADC is structurally intact, homogeneous, and highly stable, providing a robust platform for subsequent *in vitro* and *in vivo* evaluations. The controlled drug loading, preservation of antibody conformation, and long-term stability underscore the suitability of this ADC for preclinical therapeutic studies.

### 3.2 Antibody conjugation enhances paclitaxel solubility and enables physiologically compatible formulation

Free paclitaxel (PTX) is highly hydrophobic and exhibits minimal solubility in aqueous buffers, forming visible precipitates when dispersed in PBS (pH 7.4), which limits its direct use in biological studies. Conventional formulations of PTX require organic solvents or surfactants to achieve apparent solubilization; however, even under these conditions, solutions are often turbid and prone to precipitation upon dilution.

**FIGURE 3:**
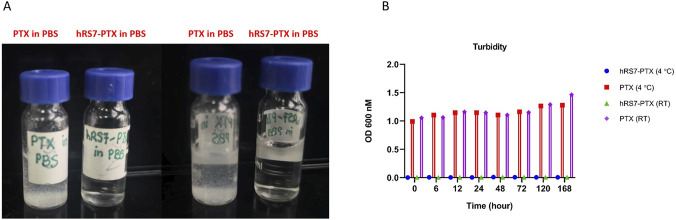
**(A)** Visual comparison of free PTX (∼435 μg/mL) and hRS7-PTX (10 mg/mL, equivalent PTX) in PBS at room temperature. Free PTX forms visible precipitates and turbid suspensions, whereas ADC solutions are clear and homogeneous. **(B)** Quantitative optical density (OD_600_) measurements at 4 °C and room temperature over 1 week. Free PTX shows high OD_600_ values reflecting poor solubility, while hRS7-PTX maintains minimal OD_600_, indicating stable aqueous solubilization. Data are presented as mean ± SD of three independent experiments.

To assess the impact of antibody conjugation on PTX solubility, free PTX (∼435 μg/mL) was compared with hRS7-PTX (10 mg/mL, equivalent PTX concentration) in PBS at room temperature. Visual inspection demonstrated that free PTX rapidly formed visible aggregates and remained turbid throughout the observation period, whereas hRS7-PTX formed clear and homogeneous solutions (Figure 3A).

Quantitative measurement of solution clarity by optical density at 600 nm (OD_600_) confirmed these observations. Free PTX exhibited high OD_600_ values, indicative of poor solubility and aggregation, while hRS7-PTX maintained consistently low OD_600_ values over multiple time points, reflecting stable aqueous solubilization (Figure 3B). Turbidity measurements were consistent at both 4 °C and room temperature over a 1-week period, demonstrating reproducible solubility and stability of the ADC.

The enhanced solubility of hRS7-PTX is attributed to covalent attachment of PTX to accessible cysteine residues on the antibody scaffold. The hydrophilic surface of the antibody effectively masks the hydrophobic regions of PTX, reducing drug self-association and precipitation. Furthermore, the antibody provides steric stabilization that prevents aggregation and enables prolonged storage at 4 °C without loss of solubility.

These findings collectively demonstrate that conjugation to hRS7 confers physiologically compatible aqueous solubility to PTX, eliminating the need for organic co-solvents or surfactants. This property facilitates consistent delivery of the full drug dose in both *in vitro* and *in vivo* studies, overcoming a major limitation of free paclitaxel. Previous work using the same PEGylated VK linker has shown that PEGylation can improve the hydrophilicity and stability of hRS7-based ADCs with hydrophobic payloads (Long et al., 2025), although the specific contribution of PEGylation in hRS7-PTX was not directly evaluated in this study.

### 3.3 hRS7-PTX exhibit potent, target-dependent *in vitro* cytotoxicity and efficient cellular internalization

**FIGURE 4:**
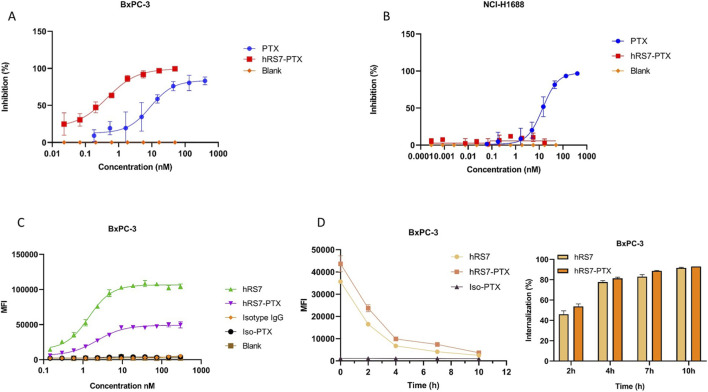
**(A)** Dose-dependent cytotoxicity of hRS7-PTX and free PTX in TROP2-positive BxPC-3 cells. hRS7-PTX exhibited markedly lower IC_50_ (0.4532 nM) compared with free PTX (9.085 nM), indicating enhanced potency. **(B)** Cytotoxicity of hRS7-PTX and free PTX in TROP2-negative NCI-H1688 cells. Negligible cytotoxicity was observed for hRS7-PTX, while free PTX exhibited baseline toxicity (IC_50_ = 13.71 nM), demonstrating target-dependent activity. **(C)** Dose-dependent binding of hRS7-PTX to TROP2-positive BxPC-3 cells, confirming preserved antigen recognition. An unpaired two-tailed t-test was used to compare maximal MFI between hRS7-PTX and unconjugated hRS7. **(D)** Internalization of hRS7-PTX in BxPC-3 cells. Left: Mean fluorescence intensity (MFI) of surface-bound ADC over time, showing progressive decrease due to internalization. Right: Percentage of internalized ADC versus time, illustrating rapid uptake within 2–4 h, confirming efficient delivery of PTX to intracellular compartments. Two-way ANOVA with Tukey’s multiple comparisons test was used to compare groups (*p < 0.05, **p < 0.01, ***p < 0.001, ****p < 0.0001; ns: not significant). Data represent mean ± SD of triplicate experiments.

The cytotoxic activity of hRS7-PTX was evaluated in BxPC-3 (TROP2-positive) and NCI-H1688 (TROP2-negative) cancer cell lines (Shao et al., 2020) and compared with free paclitaxel (PTX). In BxPC-3 cells, hRS7-PTX exhibited potent, dose-dependent cytotoxicity, with an IC_50_ of 0.4532 nM, markedly lower than free PTX (IC_50_ = 9.085 nM), indicating an approximately 20-fold enhancement in potency due to antibody-mediated delivery (Figure 4A). In NCI-H1688 cells, ADC treatment resulted in negligible cytotoxicity, whereas free PTX retained non-specific toxicity (IC_50_ = 13.71 nM), highlighting the selective, target-dependent cytotoxicity of the ADC (Figure 4B). Binding assays confirmed that hRS7-PTX retained dose-dependent binding to BxPC-3 cells, with an EC_50_ of 2.306 nM compared with 1.413 nM for the unconjugated antibody, and isotype controls showed no significant binding, indicating that conjugation did not impair antigen recognition (Figure 4C). At the highest concentrations tested, the maximal MFI of hRS7-PTX was significantly lower than that of unconjugated hRS7 (unpaired two-tailed t-test, p = 0.0007), indicating a modest decrease in binding signal after conjugation. Such small reductions are common for ADCs and are usually attributed to minor changes in epitope accessibility or hydrophobicity rather than loss of antigen recognition (Acchione et al., 2012; Buecheler et al., 2020), and the slightly higher EC_50_ of hRS7-PTX is consistent with only a modest reduction in apparent binding potency, with overall antigen binding largely preserved. Cellular internalization studies demonstrated rapid, time-dependent uptake of hRS7-PTX by BxPC-3 cells, as evidenced by a progressive decrease in surface antibody fluorescence (mean fluorescence intensity, MFI) and a corresponding increase in internalization percentage over time. Two-way ANOVA with Tukey’s multiple comparisons test showed no significant difference in surface MFI between hRS7-PTX and hRS7 at time 0, indicating comparable initial binding before internalization (Figure 4D). Internalization was rapid, with a substantial fraction of the ADCs internalized within 2–4 h, ensuring efficient delivery of PTX to intracellular compartments where it can exert cytotoxic effects.

Collectively, these results indicate that hRS7-PTX combines stable aqueous solubility, efficient target-specific binding, and rapid internalization to achieve enhanced cytotoxicity in antigen-positive cells while sparing non-target cells, providing a robust platform for selective intracellular delivery of paclitaxel.

### 3.4 ADC-mediated delivery enhances *in vivo* antitumor efficacy with minimal systemic toxicity

The *in vivo* antitumor efficacy of hRS7-PTX was evaluated in NOD/SCID mice bearing subcutaneous BxPC-3 xenografts. When tumors reached approximately 170 mm^3^, mice were randomized into treatment groups receiving hRS7-PTX, free PTX formulated with minimal DMSO/Tween-20 co-solvents (PTX) in PBS, and PBS vehicle at equimolar PTX concentrations. Treatments were administered via intravenous injection twice weekly (every 3 days) over the course of the study.

**FIGURE 5:**
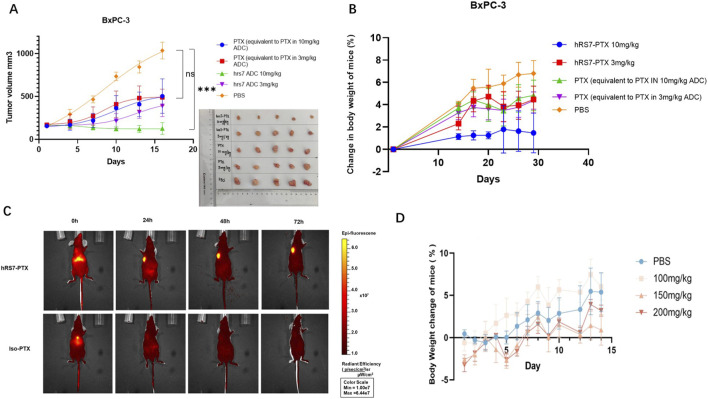
**(A)** Tumor growth curves and representative tumor images of BxPC-3 xenograft-bearing mice treated with PBS (vehicle), free PTX, and hRS7-PTX. hRS7-PTX achieving ∼75% reduction in tumor volume at the study endpoint and partial regression in several mice. Data represent mean ± SD (n = 5 mice per group). Tumor growth curves were analyzed by two-way ANOVA with Tukey’s multiple comparisons test (*p < 0.05, **p < 0.01, ***p < 0.001, ****p < 0.0001; ns: not significant). **(B)** Body weight changes of mice during treatment. No significant weight loss or adverse effects were observed in any treatment group, indicating tolerability of hRS7-PTX at the selected dosing regimen. **(C)** Longitudinal IVIS imaging confirmed selective tumor accumulation of hRS7-PTX over isotype control ADC (Iso-PTX), which showed weaker and diffuse signal distribution. **(D)** Maximum tolerated dose (MTD) studies showed that hRS7-PTX was well tolerated up to 200 mg/kg, with no mortality or ≥20% body weight loss. Data represent mean tumor volume ± SD (n = 3 per group).

Tumor growth monitoring revealed that free PTX exhibited only minimal inhibition of tumor progression, with tumors continuing to grow steadily over time. In contrast, hRS7-PTX treatment significantly suppressed tumor growth, resulting in substantially smaller tumor volumes compared with free PTX- and vehicle-treated mice. By the end of the study, tumors in ADC-treated animals were reduced by approximately 75% relative to free PTX-treated controls, and partial tumor regression was observed in several mice (Figure 5A).

Body weight measurements during the study showed no significant differences among ADC-treated, free PTX-treated, and vehicle-treated groups. No signs of overt toxicity, including lethargy, ruffled fur, or abnormal behavior, were observed (Figure 5B), indicating that hRS7-PTX was well tolerated at the selected dosing regimen.

Longitudinal *in vivo* fluorescence imaging (IVIS) was performed to assess tumor-selective accumulation of ADCs. hRS7-PTX displayed markedly higher and more sustained fluorescence at tumor sites compared with the isotype control ADC (Iso-PTX), which showed weaker and more diffuse distribution (Figure 5C). Quantitative fluorescence analysis confirmed enhanced tumor localization and prolonged retention over time, demonstrating the efficacy of antibody-mediated targeting *in vivo*.

The enhanced antitumor efficacy of hRS7-PTX is likely attributable to a combination of improved aqueous solubility, efficient tumor-targeted delivery via the antibody scaffold, and intracellular release of PTX following ADC internalization. By limiting nonspecific distribution and rapid clearance, the ADC achieves higher effective drug concentrations at the tumor site, thereby improving therapeutic outcomes.

Prior to these efficacy studies, the maximum tolerated dose (MTD) of hRS7-PTX was determined in healthy NOD/SCID mice to establish a safe dosing range. The ADC was well tolerated at doses up to 200 mg/kg, with no treatment-related mortality or body weight loss exceeding 20%, confirming a favorable safety margin and supporting the selected dosing regimen for efficacy studies (Figure 5D).

Collectively, these findings demonstrate that hRS7-PTX enables targeted, effective, and well-tolerated delivery of PTX *in vivo*, providing a promising strategy for tumor-specific chemotherapy.

## 4 Discussion

The clinical use of paclitaxel (PTX) has long been constrained by its poor aqueous solubility, nonspecific distribution, and dose-limiting toxicities. While various targeted delivery approaches, including liposomal formulations, polymer-drug conjugates, and nanoparticle carriers, have been developed to address these issues, each faces inherent limitations. Liposomes and polymer-based systems can improve solubility but often exhibit instability, suboptimal tumor accumulation, or rapid clearance. Nanoparticles may enhance tumor penetration, yet their biodistribution is often unpredictable, and formulation complexity limits scalability. These limitations highlight the persistent need for a delivery platform that can simultaneously improve solubility, ensure tumor-targeted delivery, and maintain a favorable safety profile.

In this context, our study demonstrates that PTX conjugation to antibodies overcomes several critical barriers associated with both free PTX and alternative delivery systems. PTX-ADC showed excellent solubility in aqueous buffers without the need for organic solvents, a feature that remains incompletely addressed by many existing targeted delivery platforms. Previous work using the same PEGylated VK linker in hRS7-based ADCs has shown that PEGylation can improve hydrophilicity, biophysical stability, and pharmacokinetics for hydrophobic payloads, and our findings are consistent with these observations (Long et al., 2025). This improved solubility, coupled with antibody-mediated targeting, allowed for selective cytotoxicity in antigen-positive cell lines while sparing antigen-negative controls. Flow cytometry-based internalization assays confirmed rapid uptake into target-expressing cells, ensuring efficient intracellular delivery of PTX and explaining the enhanced *in vitro* cytotoxicity compared with free drug.

The *in vivo* efficacy further supports the advantages of ADC-mediated delivery. hRS7 ADC markedly suppressed tumor growth in BxPC-3 xenografts relative to equimolar doses of free PTX, without causing weight loss or observable systemic toxicity. Consistent with previous reports demonstrating the therapeutic feasibility of paclitaxel-based ADCs, our findings further underscore how antibody conjugation can improve the therapeutic index of paclitaxel by modulating both its biological distribution and formulation behavior. These findings indicate that ADCs improve the therapeutic index by concentrating the cytotoxic payload at the tumor site while minimizing off-target exposure, a challenge that has limited the success of other targeted platforms. Notably, the combined enhancement of aqueous compatibility and tumor-specific delivery highlights the potential of ADC-based strategies to address key limitations of conventional paclitaxel formulations and existing targeted delivery platforms, addressing gaps left by existing delivery technologies.

Despite these promising results, several aspects warrant further investigation. Detailed pharmacokinetic and biodistribution studies are needed to quantify systemic exposure and tumor accumulation. Long-term safety assessments in higher animal models will also be critical for translational development. Future studies may explore alternative linker chemistries or antibody formats to optimize intracellular drug release and broaden applicability to additional tumor types or hydrophobic drugs. In addition, our *in vitro* work in this study was limited to a single TROP2-positive (BxPC-3) and TROP2-negative (NCI-H1688) cell line pair. However, in our previous work using the same hRS7-PTX ADC, we evaluated a broader panel of cell lines with differing TROP2 expression levels (Shao et al., 2020), and future studies will further extend such analyses for hRS7-PTX.

In conclusion, PTX-ADCs provide an effective strategy to address the dual challenges of poor aqueous solubility and nonspecific distribution that limit conventional paclitaxel formulations. By integrating improved aqueous compatibility, tumor-targeted delivery, and efficient intracellular release, ADCs achieve enhanced *in vitro* and *in vivo* efficacy while maintaining a favorable safety profile. These outcomes reinforce the translational prospective of ADC-based delivery for hydrophobic chemotherapeutics and support further development of paclitaxel conjugates as an advancement over existing formulation and targeting strategies.

## Data Availability

The original contributions presented in the study are included in the article/supplementary material, further inquiries can be directed to the corresponding authors.
